# Dust Exposure and Respiratory Health Among Workers in Primary Coffee Processing Factories in Tanzania and Ethiopia

**DOI:** 10.3389/fpubh.2021.730201

**Published:** 2021-09-20

**Authors:** Magne Bråtveit, Samson Wakuma Abaya, Gloria Sakwari, Bente E. Moen

**Affiliations:** ^1^Department of Global Public Health and Primary Care, University of Bergen, Bergen, Norway; ^2^Department of Preventive Medicine, School of Public Health, Addis Ababa University, Addis Ababa, Ethiopia; ^3^School of Public Health and Social Sciences, Muhimbili University of Health and Allied Sciences, Dar es Salaam, Tanzania; ^4^Department of Global Public Health and Primary Care, Centre for International Health, University of Bergen, Bergen, Norway

**Keywords:** coffee workers, dust exposure, respiratory symptoms and lung function, endotoxin, exposure assessment

## Abstract

**Introduction:** In primary coffee factories the coffee beans are cleaned and sorted. Studies from the 80- and 90-ties indicated respiratory health effects among the workers, but these results may not represent the present status. Our aim was to review recent studies on dust exposure and respiratory health among coffee factory workers in Tanzania and Ethiopia, two major coffee producing countries in Africa.

**Methods:** This study merged data from cross-sectional studies from 2010 to 2019 in 4 and 12 factories in Tanzania and Ethiopia, respectively. Personal samples of “total” dust and endotoxin were taken in the breathing zone. Chronic respiratory symptoms were assessed using the American Thoracic Society (ATS) questionnaire. Lung function was measured by a spirometer in accordance with ATS guidelines.

**Results:** Dust exposure among male production workers was higher in Ethiopia (GM 12 mg/m^3^; range 1.1–81) than in Tanzania (2.5; 0.24–36). Exposure to endotoxins was high (3,500; 42–75,083) compared to the Dutch OEL of 90 EU/m^3^. The male workers had higher prevalence of respiratory symptoms than controls. The highest symptom prevalence and odds ratio were found for cough (48.4%; *OR* = 11.3), while for breathlessness and wheezing the odds ratios were 3.2 and 2.4, respectively. There was a significant difference between the male coffee workers and controls in the adjusted FEV1 (0.26 l/s) and FVC (0.21 l) and in the prevalence of airflow limitation (FEV1/FVC < 0.7) (6.3 vs. 0.9%). Among the male coffee workers, there was a significant association between cumulative dust exposure and the lung function variables FEV1 and FVC, respectively.

**Conclusions:** The results suggest that coffee production workers are at risk of developing chronic respiratory symptoms and reduced lung function, and that the findings are related to high dust levels. Measures to reduce dust exposure should be targeted to factors identified as significant determinants of exposure.

## Introduction

Before coffee beans are brought to the primary coffee processing factories, they are processed at the farm to remove the outer layers of the coffee cherries. In the primary factories the beans are mechanically cleaned of debris, hulled to remove the hard cover, and then sorted by size and weight. Damaged and discolored coffee beans may also be removed by handpicking. Finally, the green coffee beans (GCB) are packed for transportation. Only a few of the primary processing factories include the roasting process. Roasting mostly takes place in the countries the coffee is exported to. Several studies describe aspects of work and health in coffee roasting facilities. Jones et al. (1) found significantly lower residual FEV1 among US workers handling green coffee, with long work duration, while in Germany, Oldenburg et al. (2) did not find an association between the level of coffee dust exposure and lung function impairment. Cross-shift reductions in lung function were found among Yugoslavian coffee workers (3). Sensitization to allergens in GCB might be one of the factors involved in workers respiratory effects, including work-related asthma (4, 5).

Only a few older studies have been conducted in primary coffee factories, although numerous workers are engaged worldwide in this part of the coffee production process. Studies in primary factories in Papua New Guinea and Uganda that processed both Arabica and Robusta coffee, showed levels of total dust exposure ranging 0.7–10 mg/m^3^ and 1–58 mg/m^3^ (6, 7). It has been indicated that the exposure to coffee dust is likely to cause acute and chronic respiratory symptoms (7, 8). A higher prevalence of acute respiratory symptoms was found among primary coffee factories workers in Uganda and Sri Lanka compared to controls (7, 8). Furthermore, reduced lung function was found among primary coffee factory workers in Papua New Guinea (6), indicating that the coffee workers might develop a non-specific chronic lung disease due to dust exposure at work. Exposure to organic dust may also lead to increased levels of fractional exhaled nitric oxide (FENO) (9, 10), and might be an indicator of airway inflammation.

Coffee types and the processing method differ between countries, and results from previous studies on dust exposure and respiratory health may not represent the status in the present coffee processing factories. The largest coffee exporting countries in 2016 were Brazil and Vietnam with over 1.5 million tons (11), while in the present study, the focus is on two of the major coffee producing countries in Africa, Ethiopia and Tanzania. Knowledge and practice regarding health and safety is marginal in many developing countries, particularly in Africa. As a result, many countries have limited legislation and few guidelines to protect workers. This is also the situation in Tanzania and Ethiopia. In both these countries, industrial activities are increasing, and the number of occupational injuries and diseases is increasing as well. There is a lack of a political mechanism that translates this information into action, as there is minor competency in occupational health among health personnel, politicians, and stakeholders. However, both these countries have started small projects on competence building in occupational health at their main universities, and the projects included in the present study have developed from this activity (12).

The main production processes are similar in Tanzania and Ethiopia. The work tasks are mainly performed by men, including reception of coffee beans from the farms, feeding of hoppers, precleaning, hulling, grading, bulking, and packing. However, primary coffee factories may also provide an extra quality check of the coffee beans, called “hand picking.” This process is performed by women only; they remove low quality, discolored beans by hand. However, there are differences in coffee types and in preprocessing of the coffee cherries at the farm before they enter the factory. These two countries were selected due to their systematic studies in coffee production, performed in cooperation with Norwegian researchers. Ethiopia and Tanzania are the world's fifth (384,000 tons) and 18th largest (48,000 tons) exporters, and number one and four in Africa, respectively. About 15 million people in Ethiopia depend on coffee production directly or indirectly for their living (13), while in Tanzania, the number of workers in the coffee sector is estimated to be above 2 million (14). The association between dust exposure and lung function was not found to be consistent when analyzing the studies from Tanzania and Ethiopia separately (15, 16) Thus, it is of interest to merge these studies to increase the study power.

The aim of this research was to review and summarize the results from studies the past 10 years on dust and endotoxin exposure, as well as on respiratory health among production workers in primary coffee factories in Tanzania and Ethiopia. Thus, the three studies from before year 2000 were not included in further analysis. We also aimed to identify determinants of dust exposure in order to suggest measures to reduce dust exposure.

## Materials and Methods

This article presents results from reanalysis of data from cross-sectional studies in primary coffee processing factories in Tanzania and Ethiopia conducted in the years 2010–2019. The included studies are on personal dust exposure in Tanzania (17) and Ethiopia (18) and respiratory health in Tanzania (15, 19–21) and Ethiopia (16, 22). Similar design and methodology were used in these studies in the two countries. Thus, personal dust samples were taken with the same sampling method, lung function was measured with identical instruments and the same standardized questionnaire were used for demographic information and chronic respiratory symptoms. When merging the data from the studies the variables from the original datasets were used with no transformation or with calculations of new variables. In both countries contextual information including characteristics of the factories, practices in processes, design of machines, and task performed by the workers during sampling was obtained by an observational checklist. The measured dust levels were presented separately for the two countries and were not merged for development of dust exposure models since there were some differences in potential determinants of dust exposure between the countries.

### Settings

**Figure 1:**
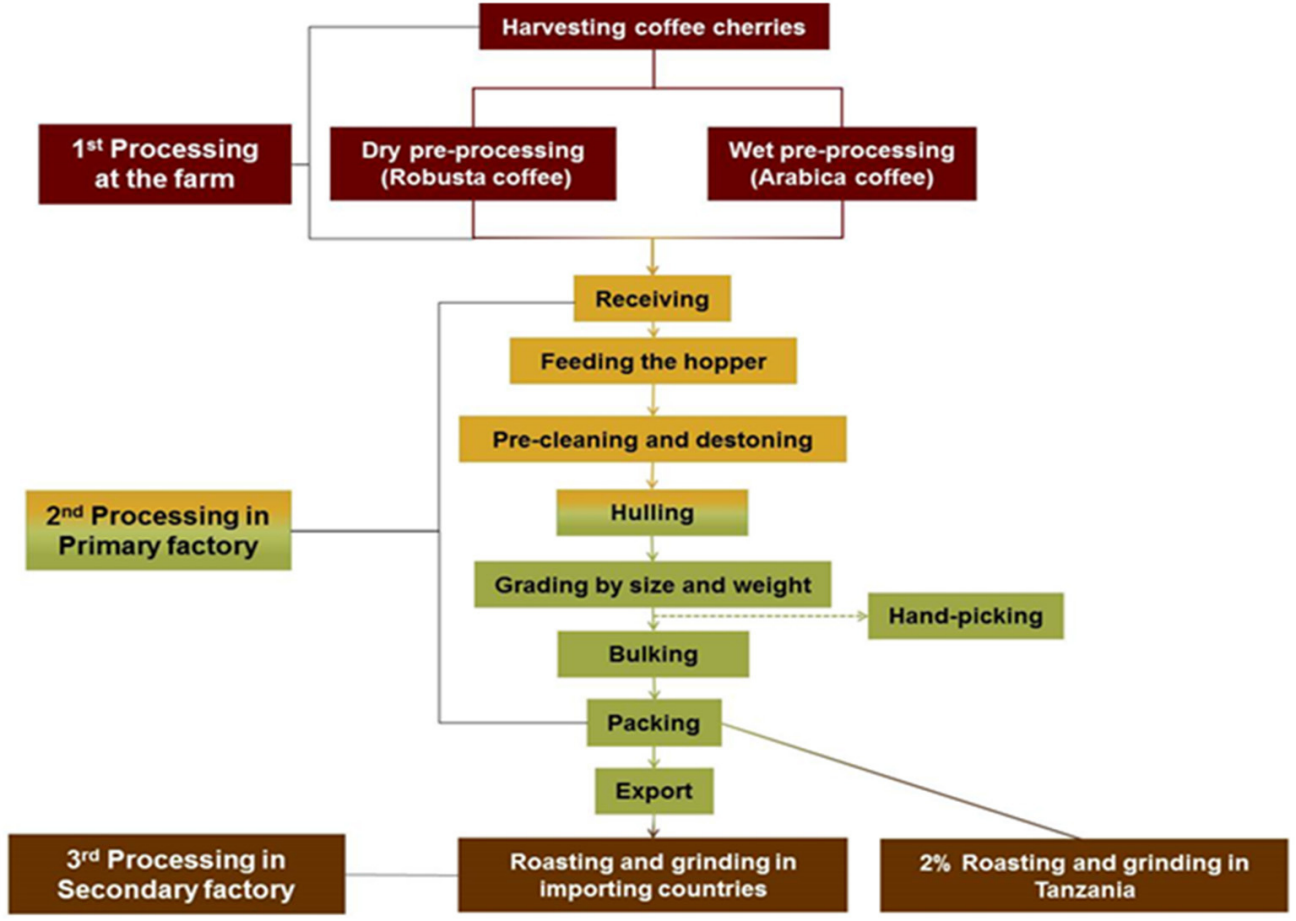
Schematic diagram for coffee processing in Tanzania (21).

The research in Tanzania was done in in four primary coffee factories, each with 30–65 production workers and an annual output of about 5,000 to 19,500 tons. In Ethiopia, 12 primary coffee processing factories were included with 60–422 production workers, and an annual production of about 1,200 to 38,000 tons. The factories and the source population were the same for all outcomes; Dust exposures, respiratory symptoms, and lung function. The main production processes are quite similar in the two countries (Figure 1). However, while Tanzania grows both Arabica and Robusta coffee types, Ethiopia produces only Arabica coffee. In Tanzania, Arabica coffee is mostly wet preprocessed at the farm whereas Robusta coffee is dry preprocessed. In Ethiopia, Arabica coffee were either dry or wet preprocessed.

### Dust and Endotoxin Measurements

Repeated personal full-shift samples of “total” dust (Tanzania; *n* = 193 and Ethiopia; *n* = 360) and endotoxin (Tanzania; *n* = 154) were taken by closed-faced 25 or 37 mm conductive cassettes at a rate of 2 l/min from the breathing zone of the production workers. Samples were analyzed gravimetrically, and a subset of samples from Tanzania was analyzed for endotoxin by kinetic chromogenic Limulus amebocyte lysate (LAL) Assay. In addition, the same methodology was used to take personal total dust samples from female hand-pickers of coffee (Tanzania; *n* = 9 and Ethiopia; *n* = 115). The results were compared to the Norwegian Occupational Exposure Limit (OEL) for organic total dust of 5 mg m^−3^ (23). For endotoxin we have used the Dutch health-based recommended occupational exposure limit of 90 EU/m^3^ as a reference value (24).

Cumulative dust in the coffee factories was calculated for each worker as a product of the geometric mean (GM) of the total dust of each respective factory and the number of seasons worked in that particular factory. Workers who had worked in coffee factories other than those included in this study had additional cumulative exposure calculated as a product of the number of seasons worked in those factories and the overall GM for total dust in the measured factories. Since identical sampling methods and strategies were used in the two countries the cumulative dust variable were merged. Cumulative dust was not calculated for the control group because these workers have different types of dust exposure.

### Respiratory Health Examinations

#### Respiratory Symptoms

We assessed chronic respiratory symptoms (yes/no) using the American Thoracic Society (ATS) standardized questionnaire among the coffee production workers from two factories in Tanzania (*n* = 140) in comparison with a control group from a beverage factory (*n* = 120) (19). The same questionnaire was used in 12 coffee factories (*n* = 115) and in three water bottling factories (*n* = 110) in Ethiopia (18).

#### Lung Function

Lung function was measured by a portable spirometer (SPIRARE 3 sensor model SPS 320) in accordance with ATS guidelines for spirometry in Tanzania (*n* = 140 coffee workers/120 controls) and Ethiopia (*n* = 115 coffee workers/110 controls) (15, 18). Of these 17 controls and 16 coffee workers were excluded from further analysis of lung function due to unacceptable spirograms. The spirometer tests were performed at any time during the day shift in all studies, and in the same time periods as the dust and endotoxin measurements. The recorded lung function parameters were; Forced expiratory volume in 1 s (FEV1 in L/s), Forced vital capacity (FVC in L) and the ratio FEV1/FVC (in %).

### Statistics

Data were analyzed by using IBM SPSS Statistics 25 for Windows, Version 25.0 (Armonk, NY: IBM Corp.). Statistical analysis was performed using Chi-square and Fischer exact test for categorical data, and independent *t*-test for continuous data. Logistic regression was used to determine odds ratio (OR) of the different respiratory symptoms (yes/no) between coffee workers (1) and controls (0) while adjusting for age (years) and current smoking (yes/no). Mixed effects models were developed for analyzing differences in lung function between coffee workers and controls, and for analyzing the association between cumulative dust exposure and lung function variables. Separate linear mixed-effects models were developed with the lung function variables FEV1, FVC, and FEV1/FVC (%) as dependent variables and age, height, current smoking, and either exposure group (coffee workers/controls) or cumulative dust exposure (in mg/m^3^^.^ year) as fixed effects. To account for repeated measurements taken in Tanzania and Ethiopia, country was viewed as a random effect. Years at school was considered as a proxy for socioeconomic status, but as it correlated significantly with age, only age was used in the models. The percentage of total variance explained by the fixed effects (age, height, current smoking, and exposure) in the respective models was calculated as the percentage change in the sum of between-country variance and within-country variances from the random model to the mixed effects model.

## Results

### Dust Exposure

Personal exposure to total dust among the coffee production workers was considerably higher in Ethiopian than in Tanzanian coffee factories (GM 12 mg/m^3^; range 1.1–81 vs. 2.5; 0.24–36) (Table 1). About 84 and 17% of the samples exceeded the OEL of 5 mg/m^3^ for total organic dust in the two countries, respectively. The majority of coffee workers did not use any type of respiratory protective devices (16, 19).

**Table 1:** Personal full-shift exposure to total dust and endotoxin among coffee production workers in Tanzania and Ethiopia.

	**Total dust (mg/m** ^**3**^ **)**					**Endotoxin (EU/m** ^**3**^ **)**				
	**Nw**	**Ns**	**AM**	**Range**	**GM (GSD)**	**Nw**	**Ns**	**AM**	**Range**	**GM × 10^**4**^ (GSD)**
**Tanzania**										
Production workers^a^	97	193	3.69	0.24–36.00	2.50 (2.44)	69	154	8,200	42–75,083	0.35 (4.36)
Arabica coffee^a^	71	124	3.69	0.24–36.00	2.10 (2.79)	43	85	3,556	42–75,083	0.14 (3.58)
Robusta coffee^a^	26	69	3.70	1.20–6.67	3.42 (1.52)^**^	26	69	13,900	1,913–46,964	1.08 (2.12)
Hand pickers^b^		9		0.3–1.7	0.9 (0.5)		9		29–372	183 (119)
**Ethiopia**										
Machine room workers^c^	60	117	17.47	1.12–77.28	12.54 (2.37)					
Transporters^c^	59	113	17.46	2.51–81.61	12.30 (2.32)					
Hand pickers^d^	60	115	1.55	0.12–9.74	1.08 (2.42)					

*Nw, number of workers sampled; Ns, number of samples*;

** *p < 0.01*.

a *Sakwari et al. (17)*.

b *Moen et al. (20)*.

c *Abaya et al. (18)*.

d *Abaya et al. (22)*.

Personal exposure to endotoxins in the Tanzanian factories was high (GM = 3,500 EU/m^3^; range 42–75,083) compared to the Dutch OEL of 90 EU/m^3^, with only two of the samples below this limit (Table 1). There was a significant correlation between exposure to total dust and endotoxin (*r* = 0.62, *P* < 0.001, *n* = 149). It was not analyzed for endotoxins in the Ethiopian factories. In Tanzania total dust and endotoxin exposures were significantly higher in Robusta than in Arabica coffee factories (Table 1), and when handling dry pre-processed coffee compared with wet pre-processed coffee (not shown). The pre-processing method of the Ethiopian Arabica coffee, dry or wet, had no impact on the exposure to total dust. The exposure for the female hand pickers did not differ between the two countries, and it was considerably lower than for the male production workers (Table 1).

### Demographic Data on Participants in the Respiratory Health studies

The studies on respiratory health among the coffee workers comprised one cross-sectional study from Tanzania and two from Ethiopia (Table 2). All coffee production workers and their respective control groups were men whereas all hand pickers and their controls were females. The response rate varied between 88 and 100% (Table 2). No difference was found between coffee workers and controls regarding weight, height, BMI, and past respiratory diseases (15, 16). In all studies the mean age among coffee workers were 4 years higher than among the controls. The controls had more education than the coffee workers. In the Tanzanian study the prevalence of current smokers among coffee workers was higher than among controls (Table 2), but the mean number of cigarettes smoked per day was low, five vs. three cigarettes per day among coffee workers and controls, respectively (15).

**Table 2:** Demographic information on the participants in the three studies of respiratory health among male coffee workers in Tanzania and Ethiopia.

	**Tanzania^a^**		**Ethiopia^b^**	
	**Controls^c^**	**Coffee workers**	**Controls^d^**	**Coffee workers**
	***n* = 120**	***n* = 140**	***n* = 110**	***n* = 115**
Response rate (%)	100	88	94	
Age (years); AM (range)	29 (19–51)	33 (19–65)^**^	31 (18–68)	35 (18–68)^**^
Years at school; AM (range)	9 (0–15)	7 (0–16)^**^	9 (0–16)	7 (0–16)^**^
Years of current work; AM (range)	5 (0.2–23)	5 (0.2–35)	3 (1–6)	7 (1–30)^**^
Current smokers; *n* (%)	14 (12)	52 (37)^**^	4 (3.6)	3 (2.6)
Cumulative dust (mg/m^3.^year); AM (range)		19 (0.5–120)		129 (4–595)

a *Sakwari et al. (15)*.

b *Abaya et al. (16)*.

c *Water bottling (n = 60) and fish factory (n = 60) workers*.

d *Water bottling workers*;

** *p < 0.01*.

### Respiratory Symptoms

When merging the studies from Tanzania and Ethiopia, the male coffee workers had higher prevalence for all recorded chronic respiratory symptoms than the controls, also when adjusting for confounders (Table 3). The highest symptom prevalence among the coffee workers was found for cough (48.4%), while the highest odds ratios were for cough and cough with sputum, followed by chest tightness, breathlessness, and wheezing (Table 3).

**Table 3:** Prevalence and odds ratio for chronic respiratory symptoms among male coffee workers and controls from Tanzania and Ethiopia.

**Chronic respiratory symptom**	**Tanzania^a^ and Ethiopia^b^**		
	**Controls^c^ *n* = 229**	**Coffee workers *n* = 252**	${\text{Odds ratio}}_{\text{adj}}^{\text{d}}$
	***n* (%)**	***n* (%)**	**OR (95% CI)^b^**
Cough	16 (7.0)	122 (48.4)^**^	11.3 (6.4–20.1)
Cough with sputum	6 (2.6)	60 (23.8)^**^	10.3 (4.3–24.6)
Breathlessness	18 (7.9)	56 (22.2)^**^	3.2 (1.8–5.7)
Chest tightness	18 (7.9)	60 (23.8)^**^	3.5 (2.0–6.3)
Wheezing	14 (6.1)	41 (16.3)^**^	2.4 (1.2–4.6)

a *Sakwari et al. (15)*.

b *Abaya et al. (16)*.

c *Water bottling and fish factory workers*.

d *Adjusted for age and current smoking*;

** *p < 0.01 in Chi-square test*.

### Lung Function

The mean FEV1, FVC, and FEV1/FVC for coffee workers were significantly lower than among controls (Table 4). In mixed effects models, adjusting for age, height, and current smoking there was still a difference between coffee workers and controls for FEV1 and FVC, but not for FEV1/FVC. The adjusted difference in FEV1 and FVC between coffee workers and controls were 0.26 l/s and 0.21 l, respectively (Table 4). The prevalence of airflow limitation (FEV1/FVC <0.7) was significantly higher (*p* = 0.002; Fischer exact test) among coffee workers (*n* = 15; 6.3%) compared to controls (*n* = 2; 0.9%).

**Table 4:** Lung function among male coffee workers in Tanzania and Ethiopia.

**Lung function variables**	**Tanzania^a^ and Ethiopia^b^**						
	**Controls**	**Coffee workers**	**Controls vs. coffee workers;** **Independent *t*-test;**	**Controls (0) vs. coffee workers (1);** **Mixed effects model^c^**			
	***n* = 213**	***n* = 239**	***p*-value**	***B***	**95%CI**		***p*-value**
FEV1, L/s, AM (SD)	3.45 (0.58)	3.26 (0.60)	0.001	−0.26	−0.38	−0.15	<0.001
FVC, L, AM (SD)	4.12 (0.70)	3.96 (0.65)	0.013	−0.21	−0.35	−0.08	0.002
FEV1/FVC, %, AM (SD)	84.0 (6.0)	82.4 (7.3)	0.009	−1.65	−3.49	0.19	0.079

a *Sakwari et al. (15)*.

b *Abaya et al. (16)*.

c *Mixed effects model with age, height, current smoking as fixed effects, and country as random effect*.

### Association Between Cumulative Dust Exposure, Lung Function, and Respiratory Symptoms

Arithmetic mean cumulative dust exposure among the male workers was 66 mg/m^3.^year (range: 0.5−595 (mg/m^3.^year)), and it was higher in Ethiopia than in Tanzania (Table 2). Table 5 shows a significant association between cumulative dust exposure and the lung function variables FEV1 and FVC among the male coffee workers. The mixed effects models adjusting for the fixed effects age, height and current smoking, indicated a significant decrease in the FEV1 and FVC of 0.9 ml/s and 0.9 ml, respectively for cumulative dust exposure of 1 mg/m^3^ per year (Table 5). This translates into an additional annual decrease in FEV1 and FVC of 15.8 ml/s and 15.8 ml, respectively for a male coffee production worker exposed to the average dust exposure in Ethiopian factories of 17.5 mg/m^3^ in a season.

**Table 5:** Linear mixed effects models for the association between cumulative dust exposure and three lung function variables among 239 male coffee workers in Tanzania and Ethiopia (random effect; country).

**Variables**	**B**	**95%CI**		***p-*value**
**FEV1 (L/s); 39.6%^a^**				
Intercept	−0.750	−2.391	0.892	0.37
Age (years)	−0.025	−0.032	−0.018	<0.001
Height (m)	2.908	1.968	3.848	<0.001
Current smoking (yes/no)	−0.030	−0.185	0.125	0.70
Cumulative dust (mg/m^3.^year)	−0.0009	−0.0018	−0.0001	0.028
**FVC (l); 31.7%^a^**				
Intercept	−1.812	−3.584	−0.042	0.045
Age (years)	−0.024	−0.032	−0.017	<0.001
Height (m)	3.940	2.941	4.938	<0.001
Current smoking (yes/no)	0.0006	−0.165	0.166	0.99
Cumulative dust (mg/m^3.^year)	−0.0009	−0.0018	−0.00002	0.046
**FEV1/FVC (%); 13.2%^a^**				
Intercept	104.725	80.036	129.414	<0.001
Age (years)	−0.145	−0.248	−0.042	0.006
Height (m)	−9.936	−24.055	4.184	0.167
Current smoking (yes/no)	−0.637	−2.962	1.689	0.59
Cumulative dust (mg/m^3.^year)	−0.010	−0.023	0.002	0.12

a *% of total variance explained by the fixed effects (age, height, and current smoking) in the respective models*.

## Discussion

The results support that there is an association between dust exposure among the male coffee production workers and respiratory health effects, including both increased prevalence of chronic respiratory symptoms and reduced lung function compared to controls. In both Tanzania and Ethiopia, a considerable fraction of the dust samples (84 and 17%) exceeded the OEL of 5 mg/m^3^ for total organic dust, and exposure to endotoxins was also high compared to the health based OEL. These results suggest that control measures should be taken to reduce dust exposure.

When analyzing the studies from Tanzania and Ethiopia separately the association between dust exposure and lung function was not consistent (15, 16) which might be due to a relatively low study power in each of the studies. In the Tanzanian study, there were no difference in the FVC and FEV1 between coffee workers and controls, as was the case in Ethiopia. After merging of the lung function data from these studies, and thereby doubling the number of study participants, the inverse relationship between cumulative dust exposure and lung function lends further support for the association between dust exposure and lung function among coffee production workers.

The lung function variables FEV1, FVC, and FEV1/FVC were all reduced among the male coffee workers compared to the controls, indicating both obstructive and restrictive lung effects. However, the significantly higher prevalence of airflow limitation (FEV1/FVC ratio < 0.70) among the coffee workers (6.3%) than the controls (0.9%) indicates that the findings mainly support an obstructive effect. Cough, wheezing, and breathlessness, symptoms that are associated with development of reduced lung function (25), had odds ratios of 11.3, 2.4, and 3.2 among the male coffee workers when compared to controls. Female hand pickers in Ethiopia were considerably less exposed, and they had lower prevalence of respiratory symptoms than the male processing workers (22). However, the female hand pickers still had higher dust exposure, a higher prevalence of almost all respiratory symptoms, and lower FEF 25–75 (0.4 l/s) than the female controls (22).

The high level of dust exposure among the coffee production workers is probably due to the open design of the process lines from manual feeding of the hopper through the machines for destoning, hulling, grading, bulking, and packing. Several of these mechanical processes have vibrating surfaces which enhance dust emission. In line with this several of the tasks performed by workers operating these machines have been identified as determinants of increased dust exposure such as feeding the hopper, grading at the gravity table, and mixing coffee (17, 18). Another important determinant of dust exposure was pouring of coffee beans from a dropping height (18). These exposure models suggest that the large variability in dust and endotoxin exposure within the exposure groups can partly be explained by difference in tasks performed by the workers. Furthermore, the identified determinants also indicates that variations in the processing methods among the factories lead to significant variability in exposure levels. For instance, the high exposure to endotoxin is associated with the dry pre-processing method used after harvest (17). Dust exposure among male production workers was higher in Ethiopian than in Tanzanian coffee factories. One reason for the difference in dust levels between the two countries might be that the Ethiopian factories were larger, with respect to both annual production rate and number of production workers. Furthermore, in Ethiopia all processing machines were situated in one hall, whereas in one half of the factories in Tanzania the machines were in different rooms. In agreement with this, Abaya et al. (18) showed that dust exposure increased with the number of coffee huller machines in the production hall. Previous old studies on total dust exposure in primary coffee processing factories presented only the range of exposure, not any central tendency of the data, which makes comparison with our studies difficult (6, 7).

The high exposure to endotoxins presumably originates from Gram-negative bacteria which have been isolated from dried and stored coffee beans (26), and might result from poor storage and drying coffee on the ground (15). Sakwari et al. (15) reported an association between exposure to cumulative exposure to endotoxin and reduced lung function among male coffee processing workers in Tanzania. Endotoxins might thus be an important constituent of the coffee dust in the development of adverse respiratory effects. However, there was no association between cumulative endotoxin exposure and asthma symptoms among the coffee workers, or any difference in FeNO levels between the coffee workers (GM = 17.4; GSD = 1.8) and controls (16.5; 1.8) (15). Furthermore, cumulative exposure to total dust or to endotoxin among the coffee workers was not associated with any significant effects on FeNO, indicating no evidence of eosinophilic airways inflammation (15). Sensitization to protein allergens in the GCB might also contribute to the respiratory effects among the coffee workers. In an Italian study the prevalence of sensitization to GCB was significantly higher in workers exposed to GCB (25.8%) than in those exposed to roasted coffee (2.7%) and in white collar workers (4.5%) (5). About 10 years ago the first coffee bean protein allergen was isolated and sequenced (27).

It is a strength of the present study that the methodology used for dust exposure, questionnaires and lung function measurements were the same in the Tanzania and Ethiopia. We used validated questionnaires and standardized methods for spirometry and dust sampling. Although questionnaire-based interviews to assess the respiratory symptoms might result in recall and interviewer bias, similar questions were used to assess the respiratory symptoms in both the coffee workers and control groups. Our analyses were adjusted for factors such as age and smoking habits, which may affect lung function. By using mixed effects models with country as random effect we also took into account possible correlation in lung function within the two countries. Although the same design and methodology were used for investigating the respective outcomes, and the same scientific environment has conducted the studies, care should still be taken when merging data from two countries. Among others there might be cultural and language differences in understanding of the chronic symptoms, differences in the impact of confounders on lung function, and in scoring of contextual information between the countries. Furthermore, weaknesses related to estimation of cumulative exposure based on current dust exposure measurements and work history includes risks of bias which may have impact on the association between exposure and lung function. The factories included in this study are considered as representative for primary coffee processing factories in the two countries in terms of size, machine types, coffee types, and design of the factories. It is difficult to know if the results are valid also in other coffee-producing countries. However, the factories studied are established in low-income countries where the competence in occupational health and safety is minor, and the results are likely to be similar in other low-income countries with a similar situation. However, since the included studies are all cross-sectional we are not able to conclude on a definite causal relationship between the dust exposure and respiratory effect. A longitudinal study should be undertaken to further support the association between dust exposure and lung function reduction, but this might be considered as unethical studies.

In conclusion the results suggest that coffee production workers are at risk of developing chronic respiratory symptoms and reduced lung function. Together with the high dust levels these findings strongly indicate that proper dust control measures are necessary to reduce the dust exposure. Personal respiratory protection which might be considered as a first approach to reduce dust exposure. However, the most effective strategy would be to reduce dust at the source by preventive measures at the machines/work tasks identified as significant determinants of increased exposure. The female hand pickers are less exposed, but they still had more symptoms than the controls, indicating that protective measures should be considered also for these workers.

## Author Contributions

MB, SA, GS, and BM contributed to conception and design of the study. SA and GS conducted the field work in Ethiopia and Tanzania, respectively. SA, GS, and MB organized the database and performed the statistical analyses. MB wrote the first draft of the manuscript. All authors contributed to manuscript revision, read, and approved the submitted version.

## Funding

This work was supported by the Norwegian Agency for Development Cooperation (NORAD) through the Norwegian Program for Capacity Building in Higher Education and Research for Development (NORHED: Number 13000646) and the Norwegian Programme for Development, Research, and Higher Education (NUFU: Number 2007/10166).

## Conflict of Interest

The authors declare that the research was conducted in the absence of any commercial or financial relationships that could be construed as a potential conflict of interest.

## Publisher's Note

All claims expressed in this article are solely those of the authors and do not necessarily represent those of their affiliated organizations, or those of the publisher, the editors and the reviewers. Any product that may be evaluated in this article, or claim that may be made by its manufacturer, is not guaranteed or endorsed by the publisher.
